# Characterization and technological functions of different lactic acid bacteria from traditionally produced Kırklareli white brined cheese during the ripening period

**DOI:** 10.1007/s12223-024-01141-8

**Published:** 2024-02-23

**Authors:** Bayram Çetin, Merve Usal, Hatice Şanlıdere Aloğlu, Annemarie Busch, Enes Dertli, Amir Abdulmawjood

**Affiliations:** 1 Department of Food Engineering, Engineering Faculty, Kırklareli University, Kırklareli, Turkey; 2 Department of Food Engineering, Chemical-Metallurgical Engineering Faculty, Yıldız Technical University, Istanbul, Turkey; 3 Institute for Food Quality and Food Safety, University of Veterinary Medicine Hannover, Foundation, Bischofsholer Damm 15, 30173 Hanover, Germany

**Keywords:** White cheese, Lactic acid bacteria, Microbial diversity, Ripening period

## Abstract

In the present study, the evolution of the physicochemical and microbiological characteristics of lactic acid bacteria (LAB) in traditional Kırklareli white brined cheese collected from 14 different cheese manufacturing facilities were investigated on different days of the 90-day ripening period. The obtained LAB within the species *Lactococcus* (*Lc*.) *lactis*, *Latilactobacillus* (*Lt*.) *curvatus*, *Lactobacillus* (*Lb*.) *casei* and *Lb*. *plantarum*, *Enterococcus* (*E*.) *durans*,* E. faecium*,* E. faecalis*, *Streptococcus macedonicus*, and *Weissella paramesenteroides* were characterized in terms of their influence on technological properties and their potential as starter cultures for traditional white brined cheese production. The results of the microbiological and physicochemical investigations showed that a few selected isolates of *Lc. lactis*, *Lb. casei*, and *Lb. plantarum* had certain functions as starter germs. Moderate acidification capacity, antibacterial activity and proteolytic activity, which are characteristic of their use as starter lactic acid bacteria, were found. Importantly, antibiotic resistance among selected *Lc. lactis*, *Lb. casei*, and *Lb. plantarum* isolates was extremely low, whereas some of these isolates demonstrated antibacterial activity against major foodborne pathogenic bacteria. Based on the results obtained in this study, selected *Lc.* and *Lb*. isolates can also be considered as starter culture in traditional cheese production.

## Introduction

Manufacturing of local dairy products is a tradition that has been preserved for centuries. The products have been characterized by great diversity, and some of them have been known since ancient times. All cheeses from a certain geographic area represent a potential national treasure and cultural heritage (Terzić-Vidojević et al. 2020).

Exemplary for this are white brined cheeses, which are produced especially in the Balkans and in Mediterranean countries such as Turkey, Egypt, and Greece. This type of cheese is matured in brine (10–18% NaCl) for a long period. The level of salt in the brine is crucial for selective properties thereof for microorganisms (Albayrak and Duran 2021). In principle, flavor, texture, and preservative properties of many fermented foods such as cheese are determined by the use of different species of the five major LAB genera: *Lactobacillus* spp., *Lactococcus* spp., *Leuconostoc* spp., *Enterococcus* spp., and *Streptococcus* spp. LAB are a heterogeneous group of gram-positive bacteria with a strictly fermentative metabolism, of which lactic acid is the most important metabolite (Temmerman et al. 2004). According to their specific roles, LAB involved in fermentation processes can be divided into two groups: starter lactic acid bacteria (sLAB) and non-starter LAB (nsLAB). sLAB may be added as starters and adjunct cultures. A starter is a culture of living microorganisms which are used to begin fermentation, producing specific changes in the chemical composition and sensory properties of the food product. On the other hand, nsLAB usually originate from the production and processing environments as spontaneous microbiota (Grujović et al. 2021).

In the traditional production method, in contrast to the basic principle using the five major LAB, a high proportion of various nsLAB can be observed during the ripening period, depending on the type of cheese. The presence of various microbial groups could influence the lipolysis and proteolysis process and therefore ultimately the ripening characteristics of the specific cheeses (Öner et al. 2006).

In traditionally produced cheeses, such as matured or fermented white cheese, a wide variety of LAB and various other specific bacteria can be found, which have a wide variety of functions or also act as indicator bacteria.

Therefore, the aim of this study was (i) to investigate the physicochemical and microbiological properties of traditional Kırklareli white brined cheese from local dairies during the 3-month ripening period, (ii) to characterize the obtained LAB and their influence on the technological properties, and (iii) to determine their potential as starter cultures for traditional cheese production.

## Material and methods

### Collection of cheese samples

In this study, Kırklareli white brined cheese samples (*n* = 56) were taken from 14 different cheese manufacturing facilities with a daily production volume varying from 10 to 70 tons of cheese in Kırklareli Province, Turkey. The samples were received prior to the packaging process and delivered to the laboratory at the Department of Food Engineering of Kırklareli University under suitable conditions. From each factory, four mold cheese samples (weighing approximately 650 g) were taken, one of which was tested for analysis directly on the first day. When the pH of the samples reached 5.0, the samples were transferred to 1 L containers and stored in their own brine at 4 °C during the ripening period. The ripening of the samples took place over a 3-month period. Physicochemical and microbiological analyses were carried out at days 1, 15, 30, and 90 of the ripening period. The overall study design is presented in the study design section.

### Physicochemical and microbiological analysis

The acidity was determined by titration, and a salt analysis was carried out on the cheese samples as described previously (Dertli et al. 2012). For the microbiological analysis, a total of 56 white cheese samples were tested for the presence of Enterobacteriaceae, *Escherichia* (*E.*) *coli*, *Staphylococcus* (*S.*) *aureus*, molds, and yeasts. For this purpose, 10 g of each cheese sample was serially diluted in 90 mL of sterile saline peptone water (0.9% NaCl, 0.1% peptone (Oxoid Ltd., Basingstoke, UK), pH 7.0) and was homogenized using a Stomacher Lab-Blender 400 (Seward Medical Ltd., London, UK). Serial dilutions were used to perform the plating procedure. The appropriate agars and incubation periods were applied as follows: Enterobacteriaceae were determined using Violet-Red-Bile-Dextrose (VRBD) Agar (Merck KGaA, Darmstadt, Germany) at 37 °C and incubated aerobically for 24–48 h (ISO 2005); total count of molds and yeasts was determined by DRBC Agar (Merck) at 25 °C and incubated aerobically for 5 days (Tournas et al. 2001); Baird Parker (BP) Agar (Oxoid) with egg-yolk tellurite addition was used to determine *S. aureus* and was incubated aerobically for 30–48 h at 35–37 °C (Tallent et al. 2001). The number of *E. coli* was determined using Tryptone Bile X-Glucuronide (TBX) Agar medium (Oxoid), and the respective agar plates were incubated for 4 h at 30 °C, followed by 18 h at 44 °C. Then, the bluish-green colonies formed after aerobic incubation were evaluated as *E. coli* (Feng et al. 2002).

### Isolation and identification of LAB and nsLAB

#### Isolation from the cheese samples

Appropriate dilution series of white cheese samples were plated out on M17 Agar (Merck) and De Man, Rogosa, and Sharpe (MRS) Agar (Merck) for enumeration of total viable LAB. The corresponding plates were incubated aerobically at 30 °C for 48 h for the growth of LAB. Different colonies were selected from the agar plates and subjected to gram-staining, cell morphology, and catalase reaction tests as described previously (Dertli et al. 2016).

#### Identification by 16S rRNA gene sequencing

After selecting the LAB isolates according to phenotypic characteristics, 31 typical isolates for each phenotype were identified as the corresponding bacterial species by sequence analysis of the 16S rRNA gene. The genomic DNA of the isolates was extracted using the Qiagen Bacterial DNA extraction kit (Vivantis Technologies Sdn Bhd, Selangor Darul Ehsan, Malaysia) in accordance with the manufacturer’s recommendations. A total of 1 μL of each genomic DNA from the isolates was used as a template for preparing each PCR reaction. Other components of the PCR approach, such as master mix and probes, had been previously prepared, and PCR analysis and sequencing were performed as described previously (Dertli et al. 2016). The obtained gene sequences were submitted to the National Center for Biotechnology Information (NCBI) BLAST database, aligned, and identified with a similarity criterion of 97–100%. Using Molecular Evolutionary Genetic Analysis (MEGA X) software, the 16S rDNA sequences of the cheese isolates were arranged to perform the phylogenetic analysis (Tamura et al. 2011). For this purpose, the neighbor-joining (NJ) method with 1000 bootstrap replicates was used and the phylogenetic tree was constructed (Saitou and Nei 1987). The partial 16S rDNA sequences of the 21 isolates identified in this study were deposited in the NCBI GenBank under accession numbers MT345607 to MT345627.

### Determination of technological properties of isolates

#### Proteolytic activity

The levels of proteolytic activity of the isolates were determined by spectrophotometric measurement of tyrosine formation. This method has been described previously (Citti et al. 1963) and was applied in this study. The spectrophotometric measurements were performed at a wavelength of 650 nm (Shimadzu UV-120–02, Kyoto, Japan). The values obtained were compared with the results of the tyrosine standard and expressed as the tyrosine equivalent (µg/mL).

#### Hydrogen sulfide production

To determine the ability of the found isolates to produce hydrogen sulfide, active cultures were cultivated in Triple Sugar Iron (TSI) Agar (Oxoid) medium and incubated for 2 weeks at 30 °C. At the end of the aerobic incubation period, a blackening of the color of the medium was observed, demonstrating the production of hydrogen sulfide (Lee and Simard 1984).

#### Lactic acid-producing abilities

The isolates should also be tested for their ability to produce lactic acid. For this purpose, 1% inoculations were performed in skimmed milk medium (Biolife Italiana S.r.l., Milan, Italy) with 18-h active cultures. This was then incubated for 6 and 24 h, respectively, at 30 °C under aerobic conditions. To determine lactic acid development, pH values were determined at the end of the incubation period. The difference between the initial pH value of skimmed milk medium and the pH value after incubation (ΔpH) was considered in the evaluation (Sarantinopoulos et al. 2001).

#### Antibacterial activities

The cheese isolates were grown in MRS broth at 37 °C for 24 h under aerobic conditions, and the culture supernatants were obtained by filtration. The inhibitory effect of H_2_O_2_ was eliminated by adjusting the pH of the supernatants to 6.5 and adding catalase reagent (5 mg/mL) (Sigma, Missouri, USA). Nutrient agar media (Oxoid) containing 18-h grown pathogenic cultures of *Bacillus cereus* FMC 19, *Escherichia coli* ATCC 25922, *Listeria monocytogenes* RSKK 472 (serovar 1/2b), *Salmonella typhimurium* NRRLE 4463, and *S. aureus* ATCC 28213 were poured into petri dishes. Then, wells of 6 mm diameter were formed on the solidified medium. A supernatant of the isolate was placed in each well to be tested for antibacterial activity. The diameters of the inhibition zones formed were recorded after a 24-h incubation period (Dertli et al. 2016).

#### Antibiotic sensitivity

Antibiotic susceptibility testing of the isolates against ampicillin (AMP, 10 µg), gentamycin (GEN, 120 µg), clindamycin (CLI, 2 µg), chloramphenicol (CHL, 30 µg), vancomycin (VAN, 30 µg), kanamycin (KAN, 30 µg), streptomycin (STR, 10 µg), erythromycin (ERY, 10 µg), and tetracycline hydrochloride (TET, 30 µg) (Bioanalyse, Ankara, Turkey) was performed by disk diffusion assay on Mueller–Hinton agar (Bioanalyse) according to EFSA (2018).

## Result and discussion

### Physicochemical analysis of cheese samples

**Fig. 1:**
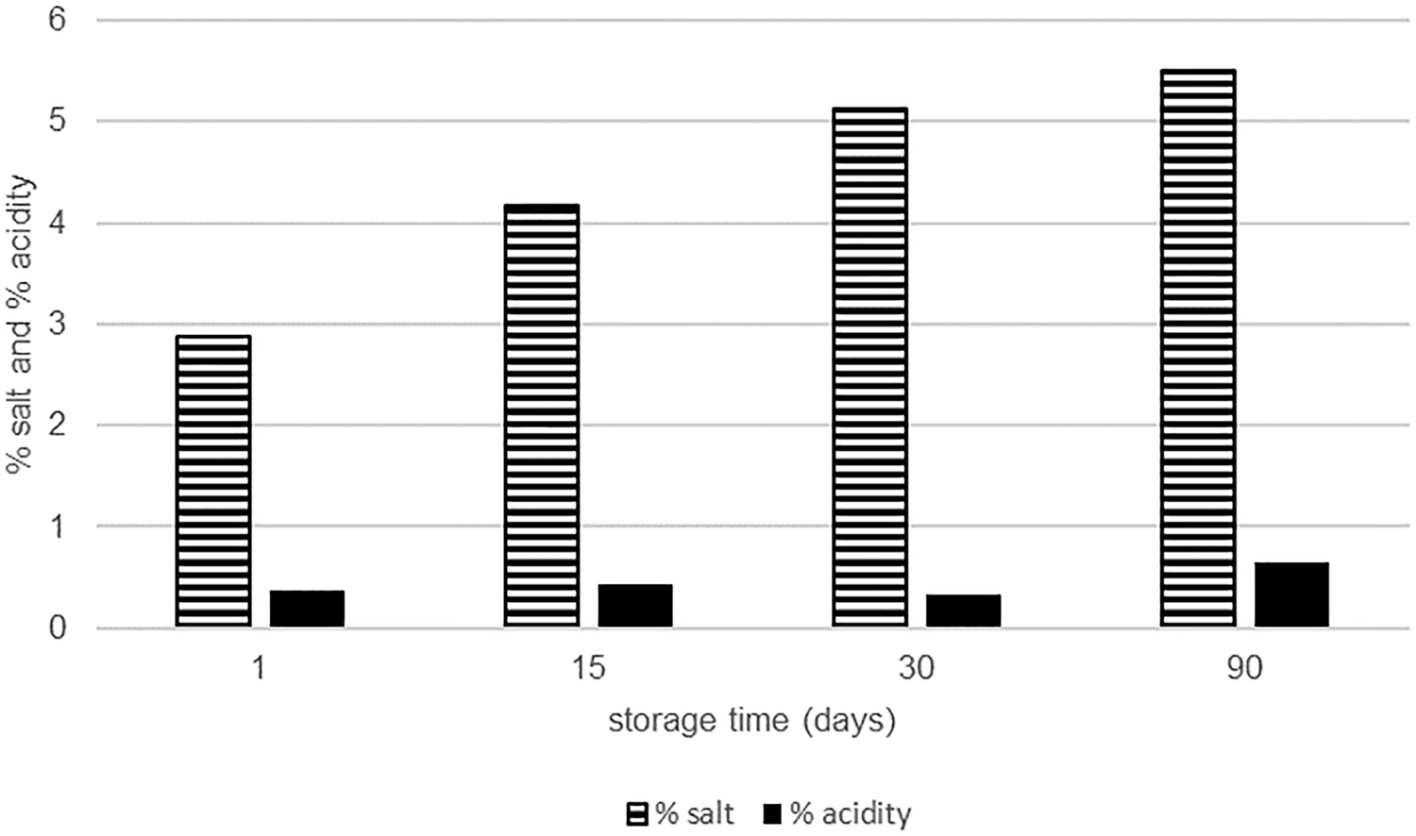
The salt and acidity levels of white cheese samples expressed as a percentage at day 90 of the ripening period

The salt and acidity values analyzed during the ripening period in traditional white cheese samples are visualized in Fig. 1. The percentage salt levels of the cheese samples were determined to be 2.88 ± 0.72%, 4.16 ± 0.84%, 5.13 ± 1.46%, and 5.51 ± 1.22% at days 1, 15, 30, and 90 of the ripening period, respectively. Similar to the salt levels of the cheese samples, there was an increase in the acidity of samples during the ripening period. Similar to the findings of this study, Uğur and Öner (2018) reported the average salt content of white cheese samples to be 4.29% and 5.59% at days 1 and 90 of the ripening period, respectively. Total titratable acidity (g lactic acid per 100 g cheese) of the samples was found to be 0.38 ± 0.18%, 0.43 ± 0.09%, 0.34 ± 0.14%, and 0.66 ± 0.14% at days 1, 15, 30, and 90 of the ripening period, respectively. However, the findings of the present study were similar to the previous findings reported for the white cheese samples collected from different regions. Hayaloglu et al. (2002) reported the titratable acidity of pickled white cheese samples to be between 0.37 and 3.80%. Çakmakçı and Kurt (1993) determined the titration acidity of fresh white cheese samples to be 0.37% and that of ripened ones to be 0.76% in their study. While the titration acidity of the cheese samples was lower on the first day, the acidity increased by the end of the ripening period. Fluctuations in titratable acidity in the later stages of ripening were caused by the formation of alkaline substances in the medium due to proteolysis during ripening and the change in dry matter (Öner and Sarıdağ 2019). It is thought that the lipolysis process, that is, the resulting fatty acid composition, also has an effect on the increase in acidity that occurs after the 90th day. Dağdemir et al. (2003) and Hayaloglu et al. (2005) also obtained similar phenomenon to our study.

### Microbiological characteristics of cheese samples

The total contamination level given in CFU per gram cheese of *E. coli*, Enterobacteriaceae, *Staphylococcus* spp., *S. aureus*, and mold-yeast was determined four times during the 90-day ripening period. All results are displayed in Table 1. At the beginning of the ripening period, a total of 6 of 14 cheese samples demonstrated *E. coli* numbers of 2–4.6 log CFU/g, while only 1 cheese sample was positive at the end of the ripening period (day 90). The initial high level of *E. coli* in cheese samples at the beginning of ripening might be a result of fecal contamination and an insufficient heating process (Beuchat and Ryu 1997). According to the Turkish Food Codex (Codex 2011), the maximum *E. coli* numbers in white cheese should be 10^2^ CFU/g. One cheese sample in this study was therefore unsuitable in terms of *E. coli* numbers. The cheese samples were also tested for the presence of Enterobacteriaceae, and counts of Enterobacteriaceae were observed between 3.5 and 6.4 log CFU/g at the first day of the ripening period (day 1). At the end of the ripening period, the lowest and highest Enterobacteriaceae numbers were 1.2 and 5.1 log CFU/g, respectively. As an indicator of the Enterobacteriaceae microbial group, high numbers might reveal poor hygiene and sanitation conditions as well as fecal contamination (Yücel and Ulusoy 2006).

**Table 1:** Numbers of *Escherichia* (*E.*) *coli*, Enterobacteriaceae, *Staphylococcus*, *Staphylococcus* (*S.*) *aureus*, and mold/yeast in traditional Kırklareli white brined cheese samples from 14 different cheese manufacturing facilities during the 90-day ripening period

		**Ripening period**																			
	**1st day**					**15th day**						**30th day**					**90th day**				
**Manufacturing facilities**	***E. coli***	**Enterobacteriaceae**	***Staphylococcus***	***S. aureus***	**Mold-yeast**		***E. coli***	**Enterobacteriaceae**	***Staphylococcus***	***S. aureus***	**Mold-yeast**	***E. coli***	**Enterobacteriaceae**	***Staphylococcus***	***S. aureus***	**Mold-yeast**	***E. coli***	**Enterobacteriaceae**	***Staphylococcus***	***S. aureus***	**Mold-yeast**
**I**	0	3.5	4.6	0	2.2		0	1.7	4.8	0	3.2	0	1.4	4.9	0	3.2	0	1.2	3.8	0	2.0
**II**	0	4.5	4.5	0	4.5		0	3.7	4.6	0	5.9	0	3.1	4.0	0	5.1	0	1.7	3.4	0	3.4
**III**	0	4.4	5.5	3.9	3.4		0	4.7	6.3	3.1	5.3	0	3.6	6.4	2.3	5.3	0	2.3	4.3	0	4.5
**IV**	0	5.7	5.7	0	3.5		0	5.0	5.9	0	5.0	0	4.2	6.4	0	4.2	0	3.1	4.9	0	3.4
**V**	2.5*	4.9	4.1	0	2.9		0	3.5	5.2	0	5.3	0	2.7	4.8	0	4.8	0	2.1	4.3	0	4.1
**VI**	2.8	6.1	6.6	4.0	4.6		0	4.8	7.0	4.8	5.5	0	3.8	6.3	5.4	7.5	0	2.2	4.8	3.4	6.1
**VII**	2	3.9	5.4	0	2.1		2.6	3.3	4.9	0	2.8	0	3.4	6.3	0	2.6	0	1.8	4.2	0	2.3
**VIII**	2.8	4.8	4.3	0	3.8		0	3.7	5.0	0	3.0	0	2.6	4.5	0	2.0	0	1.5	3.8	0	1.2
**IX**	0	5.3	4.7	3.3	3.8		0	3.1	4.6	3.2	4.3	0	3.4	5.8	3.2	4.8	0	1.3	3.2	0	4.0
**X**	0	4.4	7.2	4.2	5.9		0	3.7	6.9	3.8	7.1	0	3.6	6.3	3.4	7.0	0	2.9	3.1	2.0	6.1
**XI**	0	6.4	4.6	0	3.0		2.8	5.2	5.8	0	6.4	0	5.1	5.6	0	6.9	0	4.6	3.9	0	5.9
**XII**	4.6	5.9	6.9	5.1	5.3		4.9	6.2	7.0	5.4	7.6	3.7	4.2	6.4	5.6	6.9	3.3	5.1	4.9	3.2	5.2
**XIII**	0	5.1	6.6	3.3	4.6		0	4.3	6.7	2.9	5.7	0	3.8	6.3	2.0	5.9	0	1.6	4.8	0	5.5
**XIV**	2.5	4.9	5.4	0	4.3		2	5.3	5.4	0	4.5	0	3.8	5.4	0	4.2	0	2.5	3.4	0	2.6

^*^Values are given on average and as log CFU/g

The testing of *Staphylococcus* spp. and *S. aureus* numbers during the first day of the ripening period showed counts between 4.2 and 7.2 log CFU/g, and an approximate decrease of 1.5 log level CFU/g was observed in staphylococcus numbers at day 90. For *S. aureus*, eight samples tested negative, and in six cheese samples, *S. aureus* was observed with a contamination level of 3.3–5.1 log CFU/g. At the end of the ripening period, in three cheese samples, *S. aureus* was still observed (2.0–3.4 log CFU/g). The cell count in these was higher than the allowed detection limits of *S. aureus* according to the Turkish Food Codex (Codex 2011). Previously, higher numbers of coagulase positive *S. aureus* were reported from different regions in traditional cheese samples (Rola et al. 2016; Saka and Terzi Gulel 2018). Therefore, more attention should be paid to the milk quality by practicing more hygiene to avoid the occurrence of possible problems associated with these traditional cheese samples. Producers can select longer ripening periods recognized in the industry, such as 6 months and 1 year, as previously discussed (Öner et al. 2006).

The yeast and mold numbers in cheese samples were observed to be between 2.1–5.9 log CFU/g and 1.2–6.2 log CFU/g at days 1 and 90, respectively. These numbers were similar to previous observations (Macedo et al. 1995; Öner et al. 2006), and in general, no decrease in the yeast and mold counts during the ripening period was observed, which was in agreement with previous findings (Öner et al. 2006). The non-inhibition of the yeasts and molds in white cheese samples was associated with their potential to metabolize lactic acid, and they might also contribute to the ripening of cheese (Macedo et al. 1995). Nonetheless, in terms of white cheese, these high numbers of cells are not acceptable according to the Turkish Food Codex (Codex 2011).

### Identification of LAB and nsLAB

**Fig. 2:**
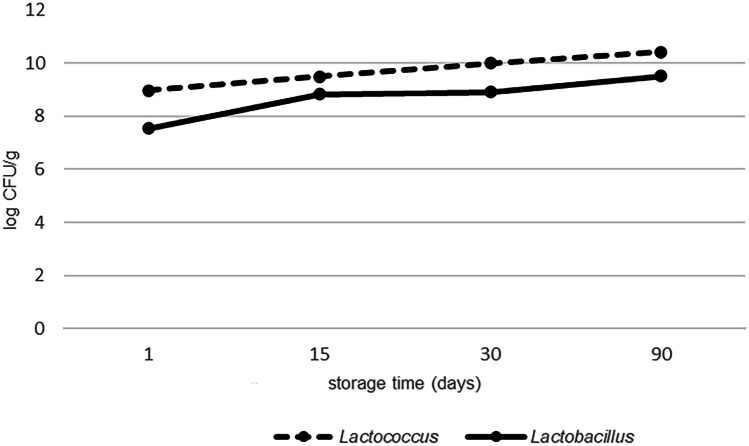
Number of lactococci and lactobacilli in white cheese samples during the ripening period

**Fig. 3:**
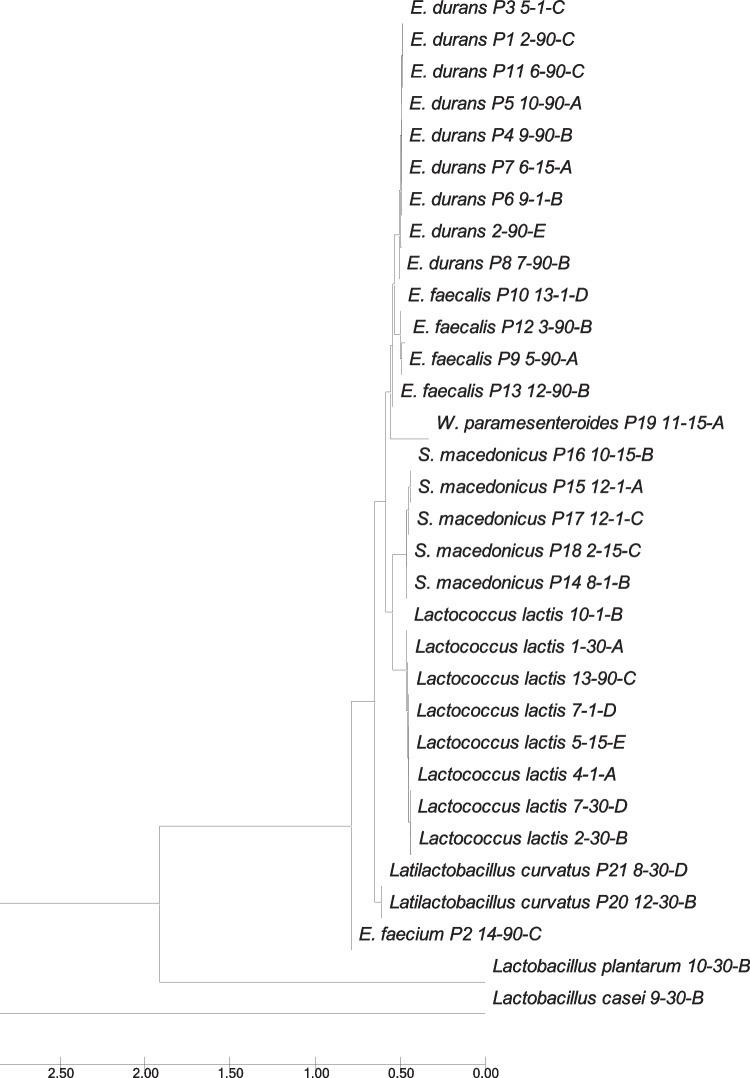
Dendrogram showing clustering of white cheese nsLAB/LAB isolates (*n* = 32) based on sequences of their 16S rRNA gene tested by neighbor-joining method

During the time of ripening, a slightly increase in the count of the LAB was observed. At the first sampling time, a mean count of 7 log CFU/g for *Lactobacillus* spp. and 9 log CFU/g for *Lactococcus* spp. was obtained. Further into the ripening phase, the numbers increased slightly and the highest counts of all microbial groups (> 10 log CFU/g) were reached at 90 days of ripening (Fig. 2). Our result was also in accordance with previous studies (Öner et al. 2006; Dertli et al. 2012). The numbers on the two different media (M17 and MRS agar) at the same sampling time were generally similar. After selecting the colonies according to phenotypic characteristics on both agars M17 and MRS, a total of 375 isolates (three to five isolates for each phenotype) were obtained from the different cheese samples during different ripening periods. We selected one isolate for each typical phenotype for further cultural characterization. Additionally, we selected a total of 51 isolates (one isolate for each typical phenotype) for further phenotypic identification (Table 2). The genotypic identification of the selected isolates (*n* = 32) by sequence analysis of the 16S rRNA gene revealed the presence of eight *Lactococcus* (*Lc.*) *lactis*, two *Latilactobacillus* (*Lt.*) *curvatus* and each one isolate of *Lactobacillus* (*Lb.*) *casei* and *Lb. plantarum*, eight *Enterococcus* (*E.*) *durans*, four *E. faecalis*, one *E. faecium*, five *Streptococcus* (*St.*) *macedonicus*, and one *Weissella* (*W.*) *paramesenteroides*, collected from traditional white cheese samples. Figure 3 demonstrates the MEGA X alignments of the 16S rRNA partial gene sequences of selected distinct LAB isolates (*n* = 32). This reveals their phylogenetic relationship, which resulted in the formation of different subgroups according to their species identification.

**Table 2:** Acidification capacities and proteolytic activity of selected isolates (*n* = 51) obtained from different ripening time of white cheese samples

**Manufactures**	**Ripening time**	**Designation of isolate**	**Δ pH 6 h**	**Δ pH 24 h**	**Production of acid (%)**	**Proteolytic activity (µg/mL)**
I	1st day	*Lactobacillus* sp. 1–1-C	0.15	1.06	0.38	83.76
	30th day	*Lactococcus lactis* 1–30-A	0.00	0.28	0.30	73.74
	90th day	*Lactococcus* sp. 1–90-D	0.00	0.16	0.45	97.59
II	15th day	*St. macedonicus* P18 2–15-C	0.13	1.28	0.48	44.65
	30th day	*Lactococcus lactis* 2–30-B	0.00	0.29	0.27	68.13
	90th day	*E. durans* P1 2–90-C	0.96	1.55	0.20	252.65
	90th day	*E. durans* 2–90-E	0.14	1.63	0.56	68.50
III	1st day	*Lactococcus* sp. 3–1-A	0.00	0.36	0.25	80.05
	30th day	*Enterococcus* sp. 3–30-B	0.00	0.98	0.49	105.31
	90th day	*E. faecalis* P12 3–90-B	0.00	1.05	0.45	106.71
	90th day	*Lactococcus* sp. 3–90-E	0.03	0.83	0.50	61.21
IV	1st day	*Lactococcus lactis* 4–1-A	0.00	0.25	0.27	167.05
	30th day	*Lactococcus* sp. 4–30-C	0.00	1.96	0.87	209.15
	90th day	*Lactobacillus* sp. 4–90-B	0.00	0.45	0.35	109.52
	90th day	*Enterococcus* sp. 4–90-E	0.23	1.82	0.40	110.22
V	1st day	*E. durans* P3 5–1-C	0.00	0.00	0.22	67.84
	15th day	*Lactococcus lactis* 5–15-E	0.00	0.42	0.23	73.04
	90th day	*E. faecalis* P9 5–90-A	0.00	0.92	0.50	76.45
VI	1st day	*Lactococcus* sp. 6–1-D	0.00	0.83	0.59	119.34
	15th day	*E. durans* P7 6–15-A	0.09	1.26	0.59	59.89
	30th day	*Lactococcus* sp. 6–30-B	0.00	1.50	0.62	143.20
	90th day	*E. durans* P11 6–90-C	0.12	1.04	0.50	66.51
VII	1st day	*Lactococcus lactis* 7–1-D	0.00	0.27	0.28	61.11
	30th day	*Lactococcus lactis* 7–30-D	0.00	2.12	0.86	61.81
	90th day	*E. durans* P8 7–90-B	0.15	0.81	0.41	57.90
	90th day	*Lactobacillus* sp. 7–90-C	0.00	1.28	0.48	529.78
VIII	1st day	*St. macedonicus* P14 8–1-B	0.06	1.18	0.46	69.82
	30th day	*Latilactobacillus curvatus* P21 8–30-D	0.06	0.06	0.27	59.89
	90th day	*Enterococcus* sp. 8–90-B	0.28	1.88	0.46	75.27
	90th day	*Lactococcus* sp. 8–90-D	0.00	1.00	0.50	66.51
IX	1st day	*E. durans* P6 9–1-B	0.00	1.39	0.49	176.88
	30th day	*Lactobacillus casei* 9–30-B	0.00	0.76	0.34	101.80
	90th day	*E. durans* P4 9–90-B	0.05	0.03	0.24	67.84
X	1st day	*Lactococcus lactis* 10–1-B	0.00	0.37	0.32	314.39
	15th day	*St. macedonicus* P16 10–15-B	0.07	1.15	0.44	55.91
	30th day	*Lactobacillus plantarum* 10–30-B	0.00	0.18	0.45	162.14
	90th day	*E. durans* P5 10–90-A	0.08	1.18	0.50	66.51
XI	1st day	*Lactococcus* sp. 11–1-D	0.70	1.67	0.36	68.13
	15th day	*W. paramesenteroides* P19 11–15-A	0.04	0.44	0.49	61.21
	90th day	*Lactobacillus* sp. 11–90-C	0.15	1.61	0.63	285.62
XII	1st day	*St. macedonicus* P17 12–1-C	0.13	1.19	0.52	69.16
	1st day	*St. macedonicus* P15 12–1-A	0.18	1.34	0.59	69.16
	30th day	*Latilactobacillus curvatus* P20 12–30-B	0.03	0.17	0.54	61.21
	90th day	*E. faecalis* P13 12–90-B	0.04	0.69	0.41	34.05
XIII	1st day	*E. faecalis* P10 13–1-D	0.11	1.23	0.56	75.12
	30th day	*Lactobacillus* sp. 13–30-C	0.00	0.51	0.30	143.20
	90th day	*Lactococcus lactis* 13–90-C	0.03	0.24	0.68	160.74
XIV	1st day	*Lactobacillus* sp. 14–1-C	0.00	0.18	0.23	129.85
	15th day	*Lactococcus* sp. 14–15-B	0.00	0.18	0.73	225.29
	30th day	*Lactococcus* sp. 14–30-B	0.30	2.08	0.47	180.88
	90th day	*E. faecium* P2 14–90-C	0.03	0.96	0.48	68.50

So far, several LAB species, especially enterococci, were reported to be present in the natural microflora of white cheese samples (Hayaloglu et al. 2002; İspirli et al. 2017; Uymaz et al. 2019). Importantly, all isolates of enterococci obtained in this study were still present in white cheese samples at day 90 of the ripening period. These observations indicate that enterococci may play a role as nsLAB in Kırklareli white cheese. Similar to our findings, it has been previously reported that *E. durans* and *E. faecalis* were dominant species in Turkish white cheese (İspirli et al. 2017). Another species isolated in the present study was *St. macedonicus*, and previous studies reported the presence of this species in different cheeses including Turkish white cheese (Lombardi et al. 2004; Ozteber and Başbülbül 2017; Uymaz et al. 2019). *St. macedonicus* was suggested to play a role in the formation of the characteristic flavor of some cheese types (Gobbetti et al. 2015) as a potential nsLAB. It should be noted that unlike previous findings, a lower level of *St. macedonicus* was present in Turkish white cheese, indicating that this bacteria may also be present in moderate amounts in Turkish white cheese (Uymaz et al. 2019). Compared to the enterococci isolates, *St. macedonicus* was present in the white cheese samples up until day 15 of ripening, which was also the case for the isolate *W. paramesenteroides* #11–15-A. Previous reports also confirmed the presence of *Weissella* species in several cheeses, including Ezine cheese (Gerasi et al. 2003; Uymaz et al. 2019).

Furthermore, within important LAB species for cheese manufacturing as starter cultures, eight isolates of *Lc. lactis*, two isolates of* Lt. curvatus*, and each one isolate of *Lb. plantarum* and *Lb. casei* were obtained from the different samples in the present study. Similar to our findings, it has been previously reported that these species were dominant starter cultures in Turkish white cheese (Ertürkmen and Öner 2015; İspirli et al. 2017). Two distinct isolates of *Lt. curvatus* (#12–30-B and #8–30-D) were isolated. Previously, *Lt. curvatus* as nsLAB and supplementary culture (Gobbetti et al. 2015) have been shown to be present in the microflora of different cheese types as well as other dairy products (Antonsson et al. 2003; Ozteber and Başbülbül 2017). The main LAB species isolated in this study were also *Lc. lactis*, confirming previous observations (Ertürkmen and Öner 2015; İspirli et al. 2017). Overall, the results of this study showed the presence of different LAB isolates, all of which could be associated with ripening of different cheese samples.

### Technological characteristics of LAB

A rich microbial diversity in LAB was observed in Kırklareli white brined cheese samples, which can originate from low pasteurization standards, as temperatures above 65 °C were not reached during the cheese production. These LAB constitute the natural starter microflora of white cheese, as no starter cultures are used in the production of Kırklareli white cheese. To understand the starter potential of LAB from white cheese, several properties of selected isolates were tested, as their moderate acid-forming ability and proteolytic activity are crucial for their use as starter cultures during white cheese production (Settanni and Moschetti 2010).

In terms of acid production, a rapid pH decline is essential to achieve adequate coagulation, curd firmness, and control of bacterial pathogen growth. The ΔpH value of the isolates, where a value of < 1 is considered low, between 1 and 1.5 is considered medium, and greater than 1.5 is considered to have a high acid-forming level (Bradley et al. 1992), was assessed. All isolates showed a low acidogenic activity in skimmed milk, with a pH decrease (ΔpH6) after 6-h incubation at 30 °C ranging from 0.00 to 0.96 pH units. Generally, acid production levels were at an intermediate level for the entire 24-h incubation period ranging from 0.00 to 2.12 pH units, and the highest acidification activity was observed in *Lc. lactis* isolate #7–30-D, suggesting their potential as starter lactic acid bacteria (LAB) (Marshall 1992; Settanni and Moschetti 2010). In this context, the acidification activity of *Lc.* and *Lb.* isolates was determined by isolate-specific characteristics, indicating their potential use as starter and/or adjunct cultures to prevent defective fermentations. The results are presented in Table 2.

Another important characteristic of the cheese isolates was the proteolytic activity, which ranged from 34.05 to 76.45%, the expression of which was determined by isolate-specific characteristics. None of the isolates was positive for the production of H_2_S. Ammar et al. (2018) categorized the proteolytic activity values of the isolates as strong, moderate, and low, with proteolytic activity values of 100–200, 50–100, and less than 50 μg tyrosine in milliliters, respectively. The results of the present study showed that all found isolates had moderate proteolytic activity except isolates *E. faecalis* #P13 12–90-B and *St. macedonicus* #P18 2–15-C, which had low levels of proteolytic activity. In general, isolates with moderate proteolytic activity should be favored for white cheese production in order to avoid the development of bitterness during ripening.

As shown in Table 3, all isolates were sensitive to most antibiotics tested in this study, and 25 different antibiotic profiles (AP1 to AP25) were observed in terms of the degree of inhibition depending on isolate-specific conditions and the antibiotics tested. For example, all isolates were strongly inhibited by ampicillin, whereas the zones of inhibition were generally lower for kanamycin and streptomycin. These two antibiotics have also been shown to be among the antibiotics to which LAB can exhibit high levels of resistance (Pesavento et al. 2014; İspirli et al. 2015). Importantly, the presence of vancomycin-resistant was noteworthy in a few isolates within the antibiotic profiles AP1, AP2, and AP3 in this study. It is worth mentioning that previous reports have documented certain isolates of enterococci derived from human feces and cheese as vancomycin-resistant (İspirli et al. 2015, 2017). Moreover, no antibiotic resistance was detected in various *St. macedonicus* isolates isolated in this study. The results presented here are consistent with previous observations demonstrating the susceptibility of *St. macedonicus* isolates (Lombardi et al. 2004). Similar to previous reports of low antibiotic resistance in *W. paramesenteroides* (Jeong and Lee 2015), the cheese isolate *W. paramesenteroides #*P19 11–15-A was not found to be resistant to the antibiotics tested. Despite the promising technological properties observed, the isolates of *Enterococcus* spp., *St. macedonicus*, and *W. paramesenteroides* found in the current study cannot be considered for incorporation into cheese production. This is because this species does not have qualified presumption of safety status due to being among the leading causes of community- and nosocomial infections (EFSA 2007). Furthermore, in contrast to previous studies showing resistance of the *Lt. curvatus* isolate to kanamycin and streptomycin (Shazali et al. 2014), the two *Lt. curvatus* isolates from white cheese samples were sensitive to all tested antibiotics, including kanamycin and streptomycin. Overall, the results of this study showed that the selected *Lc. lactis* and *Lb. plantarum* and *Lb. casei* isolates within both antibiotic susceptibility profiles AP24 and AP25 (Table 3) were not found to be resistant to antibiotics, which may be a positive feature for their use in industrial production of these cheeses.

**Table 3:** Antibiotic susceptibility profiles of the selected lactic acid bacteria isolates (*n* = 51) obtained from different ripening time of white cheese samples against nine different antibiotics

**Antibiotic profile**	No. of isolates*	**Designation of isolate**	Susceptibility profile**								
			**AMP**	**GEN**	**CLI**	**CHL**	**KAN**	**VAN**	**ERY**	**TET**	**STR**
AP1	1	*Enterococcus* sp. 4–90-E	R	R	R	R	R	R	I	S	R
AP2	1	*Enterococcus* sp. 8–90-B	R	R	R	S	R	R	I	S	R
AP3	2	*Lactobacillus* sp. 1–1-C; *Enterococcus* sp. 3–30-B	S	S	R	S	R	R	I	S	R
AP4	1	*E. durans* P11 6–90-C	S	R	R	S	R	S	R	R	R
AP5	3	*E. durans* P8 7–90-B; *Latilactobacillus curvatus* P20 12–30-B; *Lactobacillus* sp. 13–30-C	S	R	R	S	R	S	I	R	R
AP6	1	*Lactobacillus* sp. 14–1-C	S	S	S	S	R	R	S	R	R
AP7	3	*Lactococcus* sp. 6–1-D; *Lactobacillus* sp. 4–90-B; *E. faecalis* P12 3–90-B	S	S	R	S	R	S	R	R	R
AP8	2	*E. durans* P3 5–1-C; *E. durans* P4 9–90-B	S	R	R	S	R	S	R	S	R
AP9	2	*Lactococcus* sp. 3–90-E; *E. faecalis* P13 12–90-B	S	S	R	S	R	I	S	S	R
AP10	3	*E. faecalis* P9 5–90-A; *E. durans* P7 6–15-A; *St. macedonicus* P17 12–1-C	S	R	R	S	R	S	I	S	R
AP11	9	*St. macedonicus* P18 2–15-C; *E. durans* 2–90-E; *Lactococcus* sp. 6–30-B; *St. macedonicus* P14 8–1-B; *E. durans* P6 9–1-B; *St. macedonicus* P16 10–15-B; *E. durans* P5 10–90-A; *St. macedonicus* P15 12–1-A; *E. faecalis* P10 13–1-D	S	S	R	S	R	S	S	S	R
AP12	1	*Lactobacillus* sp. 7–90-C	S	S	S	S	R	R	S	S	R
AP13	1	*Latilactobacillus curvatus* P21 8–30-D	S	S	S	S	R	R	R	I	S
AP14	1	*Lactococcus* sp. 14–30-B	S	S	R	S	R	R	S	I	R
AP15	1	*E. faecium* P2 14–90-C	S	S	R	S	R	S	I	S	R
AP16	1	*Lactococcus* sp. 1–90-D	S	R	R	S	I	R	I	S	I
AP17	1	*Lactococcus* sp. 11–1-D	S	S	R	S	I	I	R	S	R
AP18	1	*Lactococcus* sp. 8–90-D	S	S	R	S	I	R	I	S	R
AP19	1	*W. paramesenteroides* P19 11–15-A	S	S	S	S	I	R	S	S	I
AP20	1	*Lactococcus* sp. 4–30-C	S	S	R	S	R	S	I	S	I
AP21	1	*Lactococcus* sp. 3–1-A	S	S	R	S	R	S	S	S	S
AP22	1	*Lactobacillus* sp. 11–90-C	S	S	S	S	I	I	S	S	R
AP23	2	*E. durans* P1 2–90-C; *Lactococcus* sp. 14–15-B	S	R	R	S	I	S	S	S	I
AP24	5	*Lactococcus lactis* 1–30-A; *Lactococcus lactis* 5–15-E; *Lactococcus lactis* 7–30-D; *Lactobacillus casei* 9–30-B; *Lactococcus lactis* 13–90-C	S	S	S	S	I	S	I	S	S
AP25	5	*Lactococcus lactis* 2–30-B; *Lactococcus lactis* 4–1-A; *Lactococcus lactis* 7–1-D; *Lactococcus lactis* 10–1-B; *Lactobacillus plantarum* 10–30-B	S	S	S	S	S	S	S	S	S

*AMP* ampicillin, *GEN* gentamycin, *CLI* clindamycin, *CHL* chloramphenicol, *KAN* kanamycin, *VAN* vancomycin, *ERY* erythromycin, *TET* tetracycline hydrochloride, *STR* streptomycin

^*^Identical susceptibility profile

**The breakpoints to discriminate between susceptible (S), intermediate (I), and resistant (R) were ≤ 21 mm, 16–20 mm, and ≥ 15 mm, respectively, for all antibiotics used in this study (according to Vlková et al. 2006)

Another important characteristic of LAB cultures from fermented food products is their antibacterial activity. The antibacterial activities of different LAB isolates were tested against important food pathogens *B. cereus* FMC 19, *E. coli* ATCC 25922, *L. monocytogenes* RSKK 472, *S. typhimurium* NRRLE 4463, and *S. aureus* ATCC 28213 (Table 4). In general, the antibacterial activity of the isolates was low, as only three of 18 isolates showed antibacterial activity (Table 4). The highest antibacterial activity was observed for the isolate *E. durans* #P3 5–1-C, as this isolate strongly inhibited *B. cereus* FMC 19, *E. coli* ATCC 25922, and *L. monocytogenes* RSKK 472. The isolate *E. durans* #P4 9–90-B similarly inhibits *E. coli* ATCC 25922 and *L. monocytogenes* RSKK 472, although this isolate was not effective against *B. cereus* FMC 19. The last isolate to show antibacterial activity in this study was *Lt. curvatus* #12–30-B, which was only effective against *L. monocytogenes* RSKK 472. Apart from these three isolates, no other 33 isolates including *Lc. lactis* and *Lb. casei* and *Lb. plantarum* showed antibacterial activity, suggesting that isolate-specific characteristics determine antibacterial activity, which may be due to the production of antibacterial substances such as bacteriocins (İspirli et al. 2017). Studies testing the genotypic and phenotypic characteristics of the bacteriocin production abilities of these isolates are still ongoing.

**Table 4:** Antibacterial activity of the selected isolates (*n* = 51) obtained from different ripening time of white cheese samples against five foodborne pathogenic bacteria

	**Isolates**	Antibacterial activity against*				
**No. of isolates**		***B. cereus* FMC19**	***E. coli* ATCC 25922**	***L. monocytogenes* RSKK 472**	***S. typhimurium* NRRLE 4463**	***S. aureus* ATCC 28213**
8	*Lactococcus lactis* 1–30-A	-	12.1	-	-	-
	*Lactococcus lactis* 13–90-C	-	11.8	-	-	-
	*Lactococcus* sp. 11–1-D	-	13.0	-	-	-
	*Enterococcus* sp. 3–30-B	16.8	-	19.2		
	*Lactococcus lactis* 7–1-D	10.2	10.5	-	-	-
	*Lactococcus* sp. 3–1-A	13.3	-	-	-	-
	*Lactococcus* sp. 6–1-D	11.1	-	11.1	-	-
	*Lactococcus lactis* 5–15-E	-	-	11.2	17.8	-
4	*E. durans* P4 9–90-B	-	26.2	16.6	-	-
	*E. durans* P1 2–90-C	11.1	-	-	-	-
	*E. durans* P3 5–1-C	17.9	23.1	17.7		
	*E. durans* P7 6–15-A	13.1	-	-	-	-
2	*E. faecalis* P12 3–90-B	11.8	-	-	-	-
	*E. faecalis* P9 5–90-A	13.5	-	11.7	-	-
4	*Lactobacillus* sp. 11–90-C	-	11.8	-	-	-
	*Lactobacillus* sp. 4–90-B	-	-	11.4	9.6	-
	*Lactobacillus* sp. 7–90-C	-	-	-	-	15.3
	*Latilactobacillus curvatus* P20 12–30-B	-	-	21.1	-	-

All other remain isolates (*n* = 33) were consistently negative against all pathogenic strains

^*^ inhibition zone (mm), - not detectable

## Conclusion

This study characterized the physicochemical and microbiological properties of traditionally produced Kırklareli white brined cheese, and the nsLAB/sLAB profile was determined during ripening. As potential starter cultures, *Lc. lactis* dominated the bacterial profile in the cheese samples, followed to a lesser extent by *Lb. casei* and *Lb. plantarum*. Worthy of mention, no antibiotic resistance was observed for any of these isolates. Moderate levels of acidifying and proteolytic activity were observed in all isolates, confirming their potential as starter culture in traditional white cheese production in the Kırklareli region. The results presented here allow a first conclusion to be drawn about the suitability of these isolated properties in cheese production, thus forming the basis for further investigations. The presence of these *Lc. lactis*, *Lb. casei*, and *Lb. plantarum* isolates as potential adjunct cultures in white cheese should be further investigated. Indeed, studies on this topic were already initiated with some of these isolates.
